# MPDRL: difficulty-aware representation learning with medical priors for vision–language pre-training

**DOI:** 10.3389/frai.2026.1906686

**Published:** 2026-09-22

**Authors:** Juntao Liu, Zhuoyi Tan, Weiqi Chen, Xiaoyong Liu

**Affiliations:** 1 School of Low-Altitude Technology and Engineering, Guangdong Polytechnic Normal University, Heyuan, China; 2 School of Computer Science, Guangdong Polytechnic Normal University, Heyuan, China

**Keywords:** contrastive learning, large language model, medical vision–language pre-training, multi-modal learning, radiology report generation

## Abstract

**Introduction:**

Medical vision-language pre-training (MedVLP) learns transferable cross-modal representations by aligning chest X-ray images with radiology reports. However, most existing CLIP-style methods have two limitations. First, they treat all unpaired image-report samples within a mini-batch as negatives, which may incorrectly penalize medically similar samples as false negatives. Second, they assume that all samples contribute equally to optimization, overlooking the heterogeneous difficulty of medical data arising from factors such as disease rarity, severity, comorbidity, and report complexity.

**Methods:**

To address these limitations, we propose Medical Prior-Driven Difficulty-Aware Representation Learning (MPDRL), which incorporates sample-level medical difficulty priors into multimodal representation learning. This method first constructs an eight-dimensional medical prior feature vector for each image-report pair to characterize sample heterogeneity. Based on these priors, it then incorporates difficulty-aware feature modulation, adaptive temperature scaling, medical prior-softened contrastive learning, and difficulty consistency regularization to reduce false-negative penalties among medically similar samples and preserve meaningful difficulty structures in multimodal representations.

**Results:**

Extensive experiments on five medical imaging datasets across three downstream tasks demonstrate the effectiveness of MPDRL. For zero-shot classification, MPDRL achieves an AUC of 87.7% on RSNA, outperforming the strongest baseline by 0.2 percentage points, and an AUC of 84.1% on CheXpert, which is 0.1 percentage points lower than the best-performing baseline, IMITATE. On CheXpert14, MPDRL achieves the best F1-score and accuracy, reaching 26.9% and 86.5%, respectively, although its AUC of 71.8% remains lower than those of several competing methods. For zero-shot image-text retrieval on CheXpert 8 × 200, MPDRL achieves Precision@1 scores of 39.9% for image-to-text retrieval and 64.7% for text-to-image retrieval. For report generation, MPDRL achieves BLEU-1 scores of 0.508 on IU X-Ray and 0.403 on MIMIC-CXR. Importantly, clinical evaluation using CheXbert and RadGraph further shows improved medical factual consistency. On IU X-Ray, MPDRL achieves CheXbert precision, recall, and F1 scores of 0.462, 0.414, and 0.437, respectively, while obtaining an RG-F1 score of 0.256. On MIMIC-CXR, it achieves CheXbert precision, recall, and F1 scores of 0.405, 0.354, and 0.378, respectively, together with an RG-F1 score of 0.238.

**Discussion:**

Overall, these results indicate that medical prior-driven difficulty modeling can improve the robustness of MedVLP representations across diverse downstream tasks.

## 1 Introduction

Medical vision-language pre-training (MedVLP) learns transferable representations by aligning medical images and free-text radiology reports within a shared embedding space. These representations can show strong transferability when they are transferred to various downstream tasks, including disease classification, image-text retrieval, and report generation (Huang et al., 2021; Zhang et al., 2022; Wang Z. et al., 2022; Tiu et al., 2022; Zhang X. et al., 2023). Most existing MedVLP approaches (Wang Z. et al., 2022; Boecking et al., 2022; Wang F. et al., 2022) adopt Contrastive Language-Image Pre-training (CLIP)-style contrastive learning (Radford et al., 2021), where matched image-report pairs are pulled closer while all other pairs within a mini-batch are uniformly treated as negatives. However, this design often performs poorly in medical scenarios. Radiology reports often contain multiple co-existing findings, uncertain observations, and complex interactions among diseases, while different patients may exhibit highly similar pathological patterns. Consequently, treating all unpaired samples as equally negative can inadvertently penalize medically related pairs and change the semantic structure of the learned representation space. Furthermore, conventional contrastive learning typically assumes that all samples contribute equally to optimization, thereby neglecting the heterogeneity inherent in medical data, including variations in disease composition, diagnostic uncertainty, rarity, severity, report complexity, image-report alignment quality, and sample representativeness. This heterogeneity is critical for medical imaging: rare or multi-abnormality samples demand more discriminative representations, uncertain or complex reports introduce semantic ambiguity, and weakly aligned image-report pairs add noise to contrastive optimization. These factors jointly compromise cross-modal alignment reliability and representation discriminability. A key question thus arises: how can medical prior knowledge about sample difficulty be effectively incorporated into vision-language representation learning without relying on hand-crafted curricula.

To address this problem, inspired by prior studies on self-paced learning and medical knowledge-guided representation learning (Jiang et al., 2015; Chen et al., 2023; Zhang et al., 2026), we propose Medical Prior-Driven Difficulty-Aware Representation Learning (MPDRL), a novel method for MedVLP. The proposed method explicitly constructs medical prior features for each image-report pair. Meanwhile, these prior features are encoded into a difficulty-aware representation, which is then directly injected into multimodal representation learning and contrastive optimization. Specifically, first, the difficulty-aware feature modulation adaptively refines visual and textual embeddings according to medical priors, enabling representations that capture clinically meaningful features. Second, the difficulty-aware temperature strategy adjusts contrastive optimization per sample, allowing heterogeneous samples to receive adaptive learning strength. Third, the medical prior-soft contrastive loss incorporates prior-based semantic similarity to reduce false negative penalties between medically similar samples.

Extensive experiments on five medical imaging datasets spanning three downstream tasks—classification, retrieval, and report generation—demonstrate that MPDRL achieves competitive performance across a range of downstream benchmarks. The proposed approach achieves strong performance on zero-shot generalization, cross-modal retrieval, and report generation. Furthermore, extensive experiments further verify that each component contributes consistently, confirming that medical prior-driven difficulty modeling, difficulty-aware representation modulation, and prior-softened contrastive learning lead to more robust, transferable vision-language representations.

## 2 Related work

### 2.1 Knowledge- and label-enhanced medical alignment

Recent studies have sought to enhance MedVLP by incorporating medical knowledge, disease labels, and report structure. MedCLIP (Wang Z. et al., 2022) reduces the impact of false negatives by decoupling image–text pairs and introducing knowledge-driven semantic matching. BioViL (Boecking et al., 2022) and BioViL-T (Guo et al., 2023) enhance domain-specific text modeling through CXR-BERT and longitudinal report information, while MGCA (Wang F. et al., 2022) performs multi-granularity alignment at the instance, token, and disease levels. MedKLIP (Wu et al., 2023) and KAD (Zhang X. et al., 2023) further introduce medical entities and structured domain knowledge to support zero-shot and few-shot diagnosis. Such representations are also beneficial for radiology report generation methods, including encoder–decoder models such as R2Gen (Chen Z. et al., 2020) and R2GenCMN (Chen et al., 2021), as well as LLM-based approaches such as R2GenGPT (Wang et al., 2023) and BootstrapLLM (Ulmer et al., 2024). However, these methods mainly focus on enhancing semantic supervision, while the heterogeneous sample-level difficulty caused by rare diseases, diagnostic uncertainty, co-morbidity, complex report semantics, or weak image–report correspondence remains insufficiently modeled.

### 2.2 Difficulty-aware and curriculum contrastive learning

To address heterogeneous sample-level difficulty, an increasing number of studies have begun to focus on difficulty-aware learning strategies. SimCLR (Chen T. et al., 2020) and MoCo (He et al., 2020) establish the instance-discrimination paradigm, in which the construction of positive and negative pairs is critical to representation learning. Supervised contrastive learning (Khosla et al., 2020) extends this paradigm by using label information to define positive and negative sets, while hard negative mining (Robinson et al., 2021) emphasizes informative negatives during optimization. Curriculum and self-paced learning (Bengio et al., 2009; Jiang et al., 2015; Kumar et al., 2010; Chen et al., 2023; Zhang et al., 2026) further suggest that organizing training samples from easy to hard can improve representation learning. However, in most general-domain studies, sample difficulty is primarily characterized by visual appearance or feature-space distance. In medical VLP, difficulty is inherently multi-factorial, involving disease co-occurrence, diagnostic uncertainty, report semantics, and image–report consistency. Existing difficulty-aware or curriculum-based strategies usually fail to jointly capture these clinically relevant factors and often rely on manually designed schedules. This motivates our medical prior-driven, difficulty-aware framework, which explicitly models sample-level medical difficulty and integrates it into both feature modulation and contrastive optimization.

### 2.3 Medical vision–language pre-training

Vision–language pre-training (VLP) has emerged as an effective paradigm for learning transferable representations by aligning visual and textual modalities (Zhang K. et al., 2023; Yin et al., 2025; Liu et al., 2024; Yin et al., 2023; Wang et al., 2025; Liang et al., 2025; Guo et al., 2023; Zheng et al., 2024). CLIP (Radford et al., 2021) demonstrated that large-scale image–text contrastive (ITC) learning can produce general-purpose visual representations with strong zero-shot transfer capability. Inspired by this success, medical VLP methods exploit paired medical images and radiology reports as a natural source of supervision. ConVIRT (Zhang et al., 2022) introduced bidirectional contrastive learning for medical image–report pairs, achieving improved performance in downstream classification and retrieval. GLoRIA (Huang et al., 2021) further modeled global–local alignment between image regions and report words for fine-grained representation learning, while CheXzero (Tiu et al., 2022) applied image–report contrastive learning to zero-shot chest X-ray (CXR) pathology classification. These methods demonstrate the effectiveness of image–report pre-training, but most of them rely on a CLIP-style objective that treats all unmatched samples within a mini-batch as negatives. This strategy can be suboptimal for medical imaging, as different patients may share similar diseases or findings, causing medically related pairs to be incorrectly pushed apart.

Recent advances in medical vision–language pre-training have increasingly moved beyond conventional image–report contrastive alignment by incorporating richer forms of medical knowledge, including anatomical structures, expert visual reasoning, and generation-aware alignment strategies (Yue et al., 2025; Liu et al., 2026b; Liang et al., 2026). CheXWorld (Yue et al., 2025) introduces a self-supervised world modeling framework for radiographic representation learning, which aims to capture multiple levels of medical knowledge beyond conventional image-level representations. Specifically, CheXWorld models local anatomical structures, global anatomical layouts, and domain variations across radiographic images. This perspective highlights the importance of learning medically meaningful visual representations that encode structural and contextual information. More recently, gaze-guided vision–language pre-training methods have explored how to incorporate radiologists' diagnostic behaviors into multimodal representation learning. CoGaze (Liu et al., 2026b) proposes a context- and gaze-guided framework for chest X-rays, where clinical context and radiologist gaze patterns are used as additional supervision signals to guide visual attention and cross-modal alignment. Such approaches emphasize that expert diagnostic processes contain valuable information beyond image-report correspondence alone. In addition to improving representation learning, recent studies have also investigated reliability issues in medical vision-language models during generation. Anatomical Region-Guided Contrastive Decoding (ARCD) (Liang et al., 2026) introduces a plug-and-play inference strategy that uses anatomical region guidance to reduce hallucinations in medical VLP models outputs. Unlike training-based alignment approaches, ARCD focuses on improving the faithfulness of generated responses by explicitly controlling the decoding process.

Different from these approaches, MPDRL focuses on another important but less explored aspect of medical VLP: the heterogeneous learning difficulty among image–report samples. Instead of assuming that all training pairs contribute equally, MPDRL constructs medical prior features from disease composition, uncertainty, rarity, co-occurrence complexity, semantic complexity, image–report alignment difficulty, and sample representativeness. These priors are further integrated into feature modulation and prior-soft contrastive learning to adaptively optimize samples with different learning difficulties.

## 3 Problem statement

Given a chest X-ray image–report dataset: $D_{M}={\left\{\left(x_{i},r_{i}\right)\right\}}_{i=1}^{N},$ where *x*_*i*_ denotes the *i*-th chest X-ray image and *r*_*i*_ denotes the corresponding radiology report, the objective of medical vision–language pre-training is to learn a visual encoder *f*_θ_(·) and a text encoder *g*_ϕ_(·) such that semantically matched image–report pairs are close in a shared embedding space, while mismatched pairs are separated. Conventional CLIP-style medical vision–language learning samples training pairs uniformly and optimizes a symmetric contrastive objective defined in Equation 1:

$$\begin{array}{l} \underset{\theta ,\phi }{\min}E_{B\sim D_{M}}\left[\frac{1}{2}\left(L_{i2t}\left(B;\theta ,\phi \right)+L_{t2i}\left(B;\theta ,\phi \right)\right)\right]. \end{array}$$ (1)

However, such a method implicitly assumes that all samples contribute equally during optimization: $P\left(\left(x_{i},r_{i}\right)\right)=\frac{1}{N},$ while samples usually exhibit substantial heterogeneity in disease composition, uncertainty, semantic complexity, visual diagnosability, and image–report alignment quality. In medical vision–language learning, these heterogeneous features not only affect optimization difficulty, but also influence the reliability of learned representations and harder cross-modal alignment. Currently, the mainstream approach to solving this problem is to incorporate medical difficulty priors into representation learning and contrastive optimization (Bengio et al., 2009; Jiang et al., 2015; Chen et al., 2023; Zhang et al., 2026). Let C = {*c*_1_, *c*_2_, …, *c*_*K*_} denote a set of disease concepts, and K denote a medical prior containing disease statistics, uncertainty cues, co-occurrence relations, semantic report structures, and sample representativeness information. For each sample (*x*_*i*_, *r*_*i*_), first construct a medical prior feature vector *F*_*i*_. Secondly, based on the medical prior K, a difficulty-aware prior encoder *h*_ψ_(·) is introduced to obtain a medical prior representation *p*_*i*_ = *h*_ψ_(*F*_*i*_) and inject the medical prior representation *p*_*i*_ into multimodal representation learning and contrastive optimization.

## 4 Our approach

As illustrated in Figure 1, the overall MPDRL consists of four major components: input and concept extraction, medical prior feature construction, prior encoding and feature modulation, and prior-soft contrastive learning.

**Figure 1:**
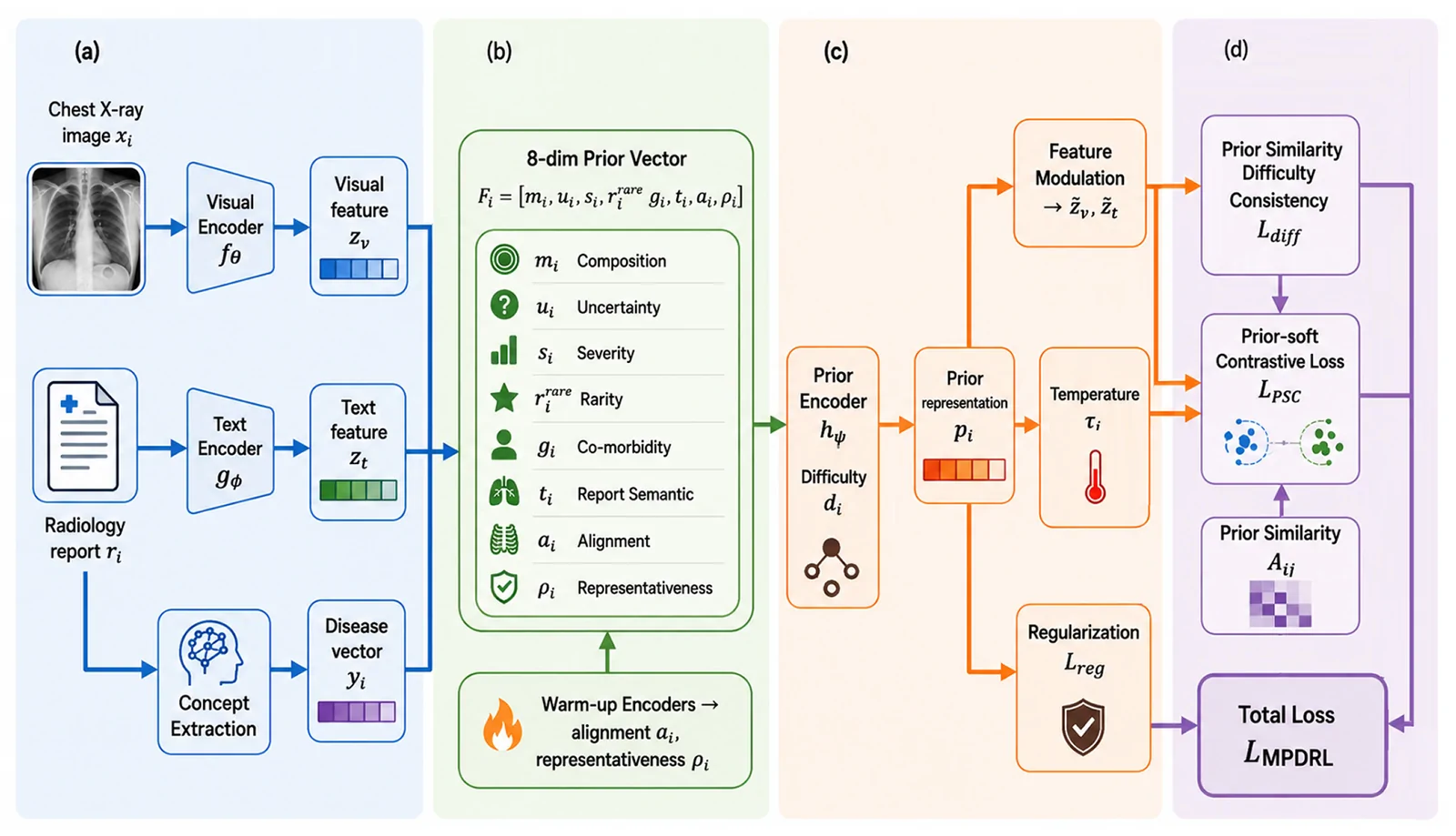
Illustration of our proposed MPDRL method, which consists of four parts, namely **(a)** input & concept extraction, **(b)** medical prior features, **(c)** prior encoding & modulation, and **(d)** prior-soft contrastive learning.

### 4.1 Medical prior-guided representation

Reports *r*_*i*_ in X-ray image–report datasets are typically free-form radiology descriptions, often involving multiple findings, uncertain observations, and complex semantic relations. To better capture such rich clinical semantics, we formulate sample representation as a multi-concept medical representation problem. Let C = {*c*_1_, *c*_2_, …, *c*_*K*_} denote a predefined set of disease concepts. For each report *r*_*i*_, we extract a multi-label disease vector: *y*_*i*_ = [*y*_*i*1_, *y*_*i*2_, …, *y*_*iK*_], where *y*_*ik*_∈{1, 0, −1}, *y*_*ik*_ = 1 indicates that disease concept *c*_*k*_ is positively mentioned, *y*_*ik*_ = 0 indicates absence or not mentioned, and *y*_*ik*_ = −1 indicates uncertainty. The label vector *y*_*i*_ can be obtained from a rule-based clinical labeler, a report label extraction model, or a prompt-based text encoder. In the prompt-based setting, the text representation of report *r*_*i*_ is: $z_{i}^{t}=g_{\phi }\left(r_{i}\right),$ and the prompt embedding for disease concept *c*_*k*_ is: *l*_*k*_ = *g*_ϕ_(Prompt(*c*_*k*_)). The semantic relevance score between report *r*_*i*_ and disease concept *c*_*k*_ is defined by Equation 2:

$$\begin{array}{l} \gamma_{ik}=\mathrm{sim}\left(z_{i}^{t},l_{k}\right)=\frac{{\left(z_{i}^{t}\right)}^{⊤}l_{k}}{\left\|z_{i}^{t}\|\|l_{k}\right\|}. \end{array}$$ (2)

Then, based on this relevance score, the disease concept vector is estimated using concept-specific thresholds, as shown in Equation 3:

$$y_{ik}=\{\begin{array}{cc} 1, & \gamma_{ik}>\delta_{k}^{+},\\ -1, & \delta_{k}^{-}\leq \gamma_{ik}\leq \delta_{k}^{+},\\ 0, & \gamma_{ik}<\delta_{k}^{-}, \end{array}$$ (3)

where $\delta_{k}^{+}$ and $\delta_{k}^{-}$ are concept-specific thresholds. This formulation, as expressed in Equation 3, allows each report to be associated with multiple disease concepts rather than a single category.

### 4.2 Medical prior features

To characterize the representation difficulty of each image–report pair (*x*_*i*_, *r*_*i*_), we construct an eight-dimensional medical prior feature vector, as defined in Equation 4:

$$\begin{array}{l} F_{i}=\left[m_{i},u_{i},s_{i},r_{i}^{\text{rare}},g_{i},t_{i},a_{i},\rho_{i}\right]. \end{array}$$ (4)

where *m*_*i*_ denotes disease composition complexity, *u*_*i*_ denotes uncertainty complexity, *s*_*i*_ denotes disease severity,$r_{i}^{\text{rare}}$ denotes disease rarity, *g*_*i*_ denotes co-morbidity complexity, *t*_*i*_ denotes report semantic complexity, *a*_*i*_ denotes image–report alignment difficulty, and ρ_*i*_ denotes sample representativeness. As shown in Algorithm 1, these features jointly encode disease composition, diagnostic uncertainty, severity, rarity, co-morbidity, report semantic complexity, image–report alignment difficulty, and sample representativeness. The design of *F*_*i*_ is motivated by the heterogeneous nature of chest X-ray image–report pairs. Samples with multiple positive findings, uncertain observations, severe or rare diseases, and uncommon co-morbidity patterns are generally more difficult to align in a shared vision–language embedding space. Therefore, disease co-occurrence statistics are first estimated over the training set to provide dataset-level medical priors, which are then used to compute co-morbidity complexity. Secondly, in addition to label-derived priors, we incorporate the representation-level difficulty cues. Report semantic complexity captures the amount of textual diagnostic information beyond disease labels, while image–report alignment difficulty is estimated using warm-up encoders *f*_θ_0__ and *g*_ϕ_0__. These warm-up encoders provide a preliminary cross-modal embedding space, allowing poorly aligned image–report pairs to be identified before the main difficulty-aware training stage. Finally, sample representativeness is measured by local density in the fused image–text embedding space. A larger ρ_*i*_ indicates that the sample lies in a dense semantic neighborhood and is therefore more typical, whereas a lower density indicates an atypical or difficult sample.

Algorithm 1 Construction of medical prior features. 
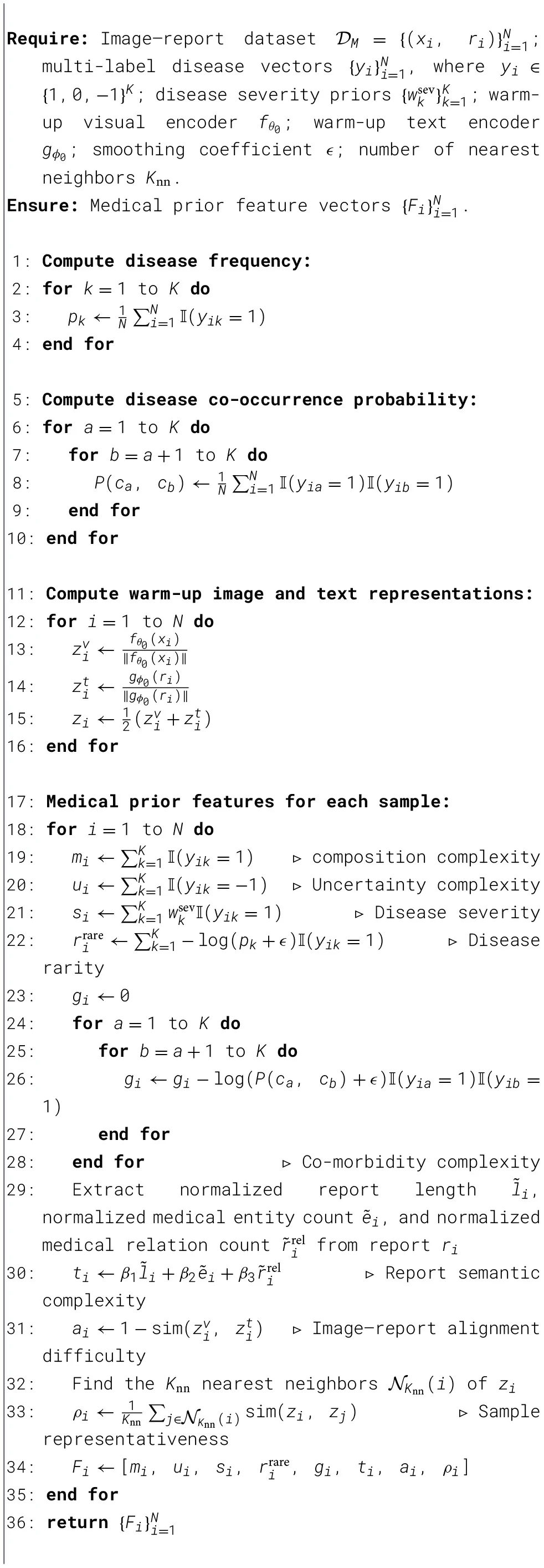

### 4.3 Medical prior-driven difficulty-aware representation learning

Based on the medical prior feature vector *F*_*i*_, we can obtain a medical prior representation as *p*_*i*_ = *h*_ψ_(*F*_*i*_), where *h*_ψ_(·) is implemented as a multi-layer perceptron. Specifically, given the normalized visual and textual representations, $z_{i}^{v}=\frac{f_{\theta }\left(x_{i}\right)}{\left|f_{\theta }\left(x_{i}\right)\right|},z_{i}^{t}=\frac{g_{\phi }\left(r_{i}\right)}{\left|g_{\phi }\left(r_{i}\right)\right|},$ we generate modality-specific modulation parameters from the medical prior representation, as formulated in Equation 5:

$$\begin{array}{l} \gamma_{i}^{v},\beta_{i}^{v}=\text{ML}{\text{P}}_{v}\left(p_{i}\right),\;\gamma_{i}^{t},\beta_{i}^{t}=\text{ML}{\text{P}}_{t}\left(p_{i}\right). \end{array}$$ (5)

The difficulty-aware visual and textual representations are then obtained through feature-wise affine modulation, as shown in Equation 6:

$$\begin{array}{l} {\tilde{z}}_{i}^{v}=\mathrm{Norm}\left(\gamma_{i}^{v}⊙z_{i}^{v}+\beta_{i}^{v}\right),\;{\tilde{z}}_{i}^{t}=\mathrm{Norm}\left(\gamma_{i}^{t}⊙z_{i}^{t}+\beta_{i}^{t}\right), \end{array}$$ (6)

where ⊙ denotes element-wise multiplication. In this process, the learned prior representation *p*_*i*_ encodes sample-specific medical difficulty information and then is used to modulate both visual and textual embeddings adaptively. Overall, Samples with higher uncertainty, stronger co-morbidity complexity, weaker image–report alignment, or rarer pathologies can be represented through adaptive feature modulation in Equation 6, leading to more medically robust multimodal embeddings.

### 4.4 Medical prior-soft contrastive learning

In medical vision–language learning, different image–report pairs often share highly similar disease semantics. Treating all non-matching pairs as strict negatives incorrectly separates medically related samples. To alleviate this issue, first, we define a medical prior similarity matrix: $A_{ij}^{K}={\mathrm{sim}}_{K}\left(y_{i},y_{j}\right),$ where $A_{ij}^{K}\in \left[0,\;1\right]$ measures semantic similarity between samples based on disease concepts, co-occurrence relations, uncertainty cues, and severity priors. Second, we define a difficulty-aware temperature parameter: τ_*i*_ = τ_0_(1+η*d*_*i*_), where $d_{i}=w^{⊤}F_{i}$ denotes the overall difficulty score derived from medical prior features. The image-to-text prior-soft contrastive loss is defined in Equation 7:

$$\begin{array}{l} \begin{array}{cc} L_{PSC}^{i2t} & =-\frac{1}{B}\overset{B}{\underset{i=1}{\sum }}\log\frac{N_{i}^{i2t}}{D_{i}^{i2t}},\\ N_{i}^{i2t} & =\exp\left({\left({\tilde{z}}_{i}^{v}\right)}^{⊤}{\tilde{z}}_{i}^{t}/\tau_{i}\right)+\underset{j\neq i}{\sum }A_{ij}^{K}\exp\left({\left({\tilde{z}}_{i}^{v}\right)}^{⊤}{\tilde{z}}_{j}^{t}/\tau_{i}\right),\\ D_{i}^{i2t} & =\overset{B}{\underset{j=1}{\sum }}\exp\left({\left({\tilde{z}}_{i}^{v}\right)}^{⊤}{\tilde{z}}_{j}^{t}/\tau_{i}\right). \end{array} \end{array}$$ (7)

Similarly, the text-to-image prior-soft contrastive loss is given by Equation 8:

$$\begin{array}{l} \begin{array}{cc} L_{PSC}^{t2i} & =-\frac{1}{B}\overset{B}{\underset{i=1}{\sum }}\log\frac{N_{i}^{t2i}}{D_{i}^{t2i}},\\ N_{i}^{t2i} & =\exp\left({\left({\tilde{z}}_{i}^{t}\right)}^{⊤}{\tilde{z}}_{i}^{v}/\tau_{i}\right)+\underset{j\neq i}{\sum }A_{ij}^{K}\exp\left({\left({\tilde{z}}_{i}^{t}\right)}^{⊤}{\tilde{z}}_{j}^{v}/\tau_{i}\right),\\ D_{i}^{t2i} & =\overset{B}{\underset{j=1}{\sum }}\exp\left({\left({\tilde{z}}_{i}^{t}\right)}^{⊤}{\tilde{z}}_{j}^{v}/\tau_{i}\right). \end{array} \end{array}$$ (8)

The final prior-soft contrastive objective is then obtained by combining the two directional losses, as shown in Equation 9:

$$\begin{array}{l} L_{PSC}=\frac{1}{2}\left(L_{PSC}^{i2t}+L_{PSC}^{t2i}\right). \end{array}$$ (9)

Compared with standard CLIP-based contrastive learning methods, the proposed objective aims to reduce false negative penalties between medically similar samples and improve semantic consistency in the learned multimodal representation space.

### 4.5 Difficulty consistency regularization

To regularize difficulty-aware representations, we maintain consistency between medical prior difficulty and multimodal embeddings. Specifically, we predict difficulty scores from visual and textual representations: ${\hat{d}}_{i}^{v}=h_{v}\left({\tilde{z}}_{i}^{v}\right),{\hat{d}}_{i}^{t}=h_{t}\left({\tilde{z}}_{i}^{t}\right).$ The difficulty consistency loss is defined by Equation 10:

$$\begin{array}{l} L_{diff}=\frac{1}{B}\overset{B}{\underset{i=1}{\sum }}\left(\left|{\hat{d}}_{i}^{v}-d_{i}|+|{\hat{d}}_{i}^{t}-d_{i}|+|{\hat{d}}_{i}^{v}-{\hat{d}}_{i}^{t}\right|\right). \end{array}$$ (10)

This regularization in Equation 10 encourages multimodal representations to preserve medically meaningful difficulty structures.

### 4.6 Overall optimization objective

The final optimization objective of MPDRL is given by Equation 11:

$$\begin{array}{l} L_{MPDRL}=L_{PSC}+\lambda_{diff}L_{diff}+\lambda_{reg}L_{reg}, \end{array}$$ (11)

where $L_{reg}={\left\|p_{i}\right\|}_{2}^{2}$ is a regularization term for medical prior representations. Therefore, the final optimization problem is formulated as Equation 12:

$$\begin{array}{l} \underset{\theta ,\phi ,\psi }{\min}E_{B\sim D_{M}}\left[L_{PSC}+\lambda_{diff}L_{diff}+\lambda_{reg}L_{reg}\right]. \end{array}$$ (12)

## 5 Experiments

### 5.1 Datasets

#### 5.1.1 Pre-training dataset

##### 5.1.1.1 MIMIC-CXR

This publicly accessible corpus pairs chest radiographs with free-text clinical reports spanning 65,379 patients, encompassing 377,110 images from 227,835 radiological studies (Johnson et al., 2019). Reports typically comprise structured fields, including indication, technique, Findings, and Impression—while images cover both frontal (PA/AP) and lateral projections. We adopt the standard partition for model training (as shown in Table 1). To maintain modality consistency during the pre-training phase, only frontal-view radiographs are retained, together with the Findings and Impression sections of the associated reports, as these two sections most directly encode image-level diagnostic content.

**Table 1:** Statistics and data splits of the datasets used for pre-training and downstream evaluation.

Dataset	Scale	Train	Val	Test
MIMIC-CXR	377,110 images	368,960	2,991	5,159
RSNA Pneumonia	29,700 images	25,184	1,500	3,000
IU-Xray	7,470 images/3,955 reports	3,164 reports	395 reports	396 reports
ChestX-ray14	112,120 images	78,468	11,219	22,433
CheXpert 8 × 200	1,600 images	–	1,600 images for evaluation only	

#### 5.1.2 Downstream datasets

For downstream evaluation, we adopt several standard benchmarks covering image classification, zero-shot recognition, image–text retrieval, and radiology report generation. Table 1 summarizes the scale and official partitions of the downstream datasets used.

##### 5.1.2.1 RSNA Pneumonia (RSNA)

Originally released by the NIH, this benchmark provides 29,700 frontal chest radiographs annotated with bounding boxes delineating pneumonia opacities, drawn from a larger pool of over 260,000 images (Shih et al., 2019). Its annotation granularity supports classification, localization, and segmentation alike. We leverage it for two evaluation protocols: zero-shot classification and linear probing, adhering to the official partition listed in Table 1.

##### 5.1.2.2 IU-Xray

This moderately sized collection comprises 7,470 chest radiographs matched with 3,955 English-language radiology reports, each containing structured *Findings* and *Impression* sections (Demner-Fushman et al., 2015). Because patients are imaged in both frontal and lateral views, the dataset facilitates cross-view descriptive analysis. Its well-organized report structure has made it a standard testbed for image-to-text generation and vision–language evaluation. Following established preprocessing protocols (Li et al., 2023; Huang et al., 2023), we randomly partition the reports into training (80%), validation (10%), and testing (10%) subsets.

##### 5.1.2.3 ChestX-ray14

Comprising 112,120 frontal-view radiographs from 30,805 patients, this large-scale benchmark assigns one or more of 14 thoracic pathology labels per image, rendering it well suited for multi-label classification (Wang et al., 2017). We evaluate on the NIH-designated test split of 22,433 images, all verified by board-certified radiologists, consistent with widely adopted evaluation conventions.

##### 5.1.2.4 CheXpert 8 × 200

This class-balanced evaluation subset samples 200 images per category across 8 prevalent thoracic conditions from the CheXpert validation set, yielding 1,600 radiographs in total (Zhang et al., 2022). It has become a de facto benchmark for zero-shot classification and cross-modal retrieval. We adopt the standard protocol: for each disease category a small query set of images and textual descriptions is selected, with all 200 class-specific images serving as retrieval candidates to assess zero-shot image–text matching performance.

##### 5.1.2.5 MIMIC-CXR

Beyond pre-training, we also utilize MIMIC-CXR as a downstream benchmark to evaluate radiology report generation, measuring both the clinical fidelity and linguistic coherence of image-conditioned textual descriptions.

### 5.2 Implementation details

We use *K* = 14 common thoracic disease concepts following the CheXpert label naming scheme. Each report *r*_*i*_ is represented as a multi-label vector $y_{i}\in {\left\{1,0,-1\right\}}^{14}$. The thresholds (δ^+^) and (δ^−^) determine whether a disease concept is considered positive, uncertain, or absent. Because these two thresholds jointly determine the resulting label distribution, we evaluate them using a two-dimensional grid satisfying (δ^−^ < δ^+^). In particular, we consider the threshold ranges shown in Equation 13:

$$\begin{array}{l} \delta^{+}\in \left\{0.15,0.2,0.25,0.30,0.35,0.40,0.45,0.5\right\}, \end{array}$$ (13)

$$\begin{array}{l} \delta^{-}\in \left\{0.10,0.15,0.20,0.25,0.30,0.35\right\}. \end{array}$$

The model remains stable over a relatively broad region. As shown in Figure 2a, the setting (δ^+^, δ^−^) = (0.30, 0.15) achieves the best zero-shot AUC and a favorable precision-recall trade-off, yielding the best validation performance. Increasing δ^+^ substantially reduces the number of positive concepts and improves precision at the expense of recall, whereas a very small δ^−^ reduces the number of uncertain labels.

**Figure 2:**
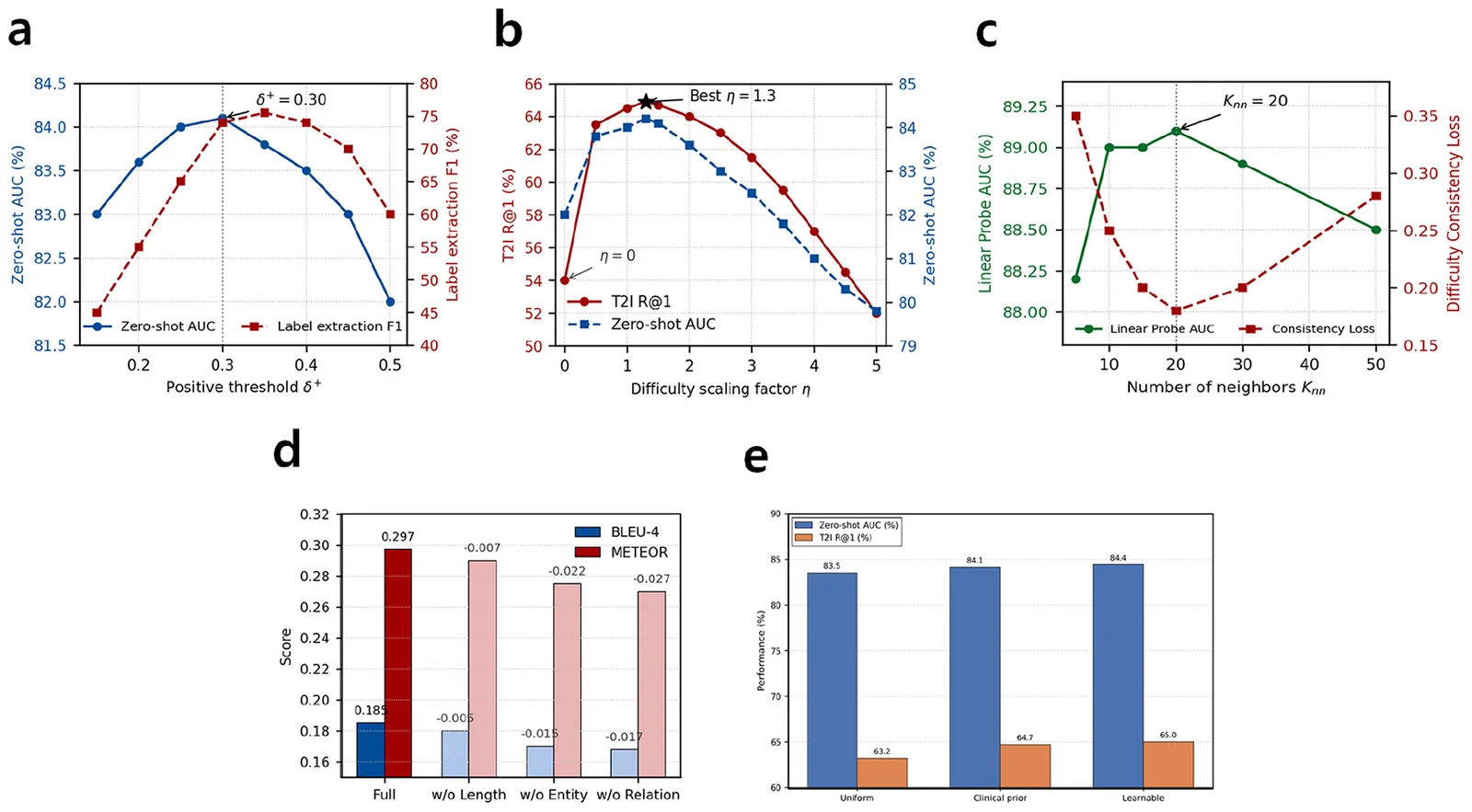
Parameter sensitivity and ablation studies. **(a)** Sensitivity to the disease concept extraction threshold δ^+^. **(b)** Sensitivity to the difficulty scaling factor η. **(c)** Effect of the k-NN graph size *K*_nn_. **(d)** Ablation on the report semantic complexity components. **(e)** Comparison of difficulty weighting strategies.

For the prior-soft contrastive loss, the similarity between samples *i* and *j* is computed using uncertainty-aware cosine similarity over their disease label vectors, weighted by inverse disease frequency. The uncertain-label coefficient λ_*u*_ controls how uncertain findings (*y*_*ik*_ = −1) are treated. Specifically, we define the uncertainty-aware label weight q_*ik*_ as in Equation 14:

$$q_{ik}=\{\begin{array}{cc} 1, & y_{ik}=1,\\ \lambda_{u}, & y_{ik}=-1,\\ 0, & y_{ik}=0. \end{array}$$ (14)

Setting λ_*u*_ = 0 treats uncertain findings as absent, whereas λ_*u*_ = 1 treats them identically to positive findings. We vary λ_*u*_ in {0.25, 0.50, 0.75, 1.00} and find that intermediate values consistently perform better, with λ_*u*_ = 0.5 providing the best balance. This supports modeling uncertain findings as partially positive semantic evidence rather than completely ignoring them or treating them as definite abnormalities. We therefore adopt λ_*u*_ = 0.5 and compute the medical prior similarity as shown in Equation 15:

$$\begin{array}{l} A_{ij}^{K}=\frac{\sum_{k=1}^{K}q_{ik}q_{jk}w_{k}^{\text{inv}}}{\sqrt{\sum_{k=1}^{K}q_{ik}^{2}w_{k}^{\text{inv}}}\sqrt{\sum_{k=1}^{K}q_{jk}^{2}w_{k}^{\text{inv}}}}, \end{array}$$ (15)

where the inverse disease frequency weight $w_{k}^{\text{inv}}$ is defined by Equation 16:

$$\begin{array}{l} w_{k}^{\text{inv}}=\log\frac{N}{1+\sum_{i}q_{ik}}, \end{array}$$ (16)

and *N* is the number of training samples. The resulting similarity matrix is clipped to [0, 1].

The sample-specific temperature is defined by Equation 17:

$$\begin{array}{l} \tau_{i}=\tau_{0}\left(1+\eta {\bar{d}}_{i}\right), \end{array}$$ (17)

with τ_0_ = 0.07 and η = 1.0. As shown in Figure 2b, difficulty-aware scaling improves over the non-adaptive baseline (η = 0), with the best result at η = 1.3 and stable performance for η∈[1.0, 2.0].

The severity prior $w_{k}^{\text{sev}}$ is assigned according to the visual and semantic difficulty of thoracic findings, with values in {0, 1, 2, 3} denoting normal, mild/common, moderate, and severe/challenging findings, respectively. By default, we adopt the severity prior vector specified in Equation 18:

$$\begin{array}{l} w^{\text{sev}}=\left[0,1,1,1,1,2,2,2,2,2,2,2,3,3\right]. \end{array}$$ (18)

To determine whether performance is driven by the exact manually assigned values, we compare the following variants:

1. **Uniform**: all disease concepts receive the same weight;
2. **Binary**: normal findings receive weight 0 and all abnormal findings receive weight 1;
3. **Ordinal**: the proposed four-level severity prior;
4. **Perturbed**: each nonzero ordinal weight is independently increased or decreased by one level and clipped to [0, 3];
5. **Permuted**: the same set of severity values is randomly reassigned across disease concepts;
6. **Learned**: severity weights are initialized using the ordinal prior and subsequently optimized with a non-negativity constraint.

The results are summarized in Table 2. The proposed ordinal prior achieves the best performance, while the perturbed and clinically grouped variants obtain comparable results. In contrast, randomly permuting the severity assignments leads to a noticeable degradation. These results indicate that MPDRL benefits from preserving a meaningful relative ordering among disease concepts rather than relying on precisely tuned numerical severity values. For example, suppose three samples contain, respectively, one mild finding, one severe finding, and one mild plus one moderate finding. Under the ordinal prior, their severity scores are *s*_1_ = 1, *s*_2_ = 3, *s*_3_ = 1+2 = 3. After feature-wise normalization, these values provide relative difficulty information rather than absolute clinical risk scores. Under the perturbed setting, changing the moderate level from 2 to 1 or 3 causes only a limited change in the final normalized difficulty score because severity is combined with seven additional prior dimensions and subsequently normalized using training-set percentiles.

**Table 2:** Sensitivity analysis of the severity prior $w_{k}^{\text{sev}}$.

Severity prior	CheXpert	CheXpert 8 × 200/report generation		
	Zero-shot AUC ↑	Retrieval R@1 ↑	Retrieval R@5 ↑	Report F1 ↑
Uniform	0.821	64.1	82.5	0.487
Binary	0.823	64.5	82.9	0.490
**Ordinal (default)**	**0.841**	**65.8**	**84.1**	**0.497**
Perturbed (±1)	0.829	65.4	83.8	0.495
Clinical grouping	0.834	65.6	83.9	0.496
Random permutation	0.817	62.7	81.0	0.481

Zero-shot AUC is evaluated on the CheXpert dataset, while Retrieval R@1, Retrieval R@5, and Report F1 are evaluated on the corresponding downstream tasks. Bold values indicate the best performance.

For the report semantic complexity score defined in Equation 19:

$$\begin{array}{l} t_{i}=\beta_{1}{\tilde{l}}_{i}+\beta_{2}{ẽ}_{i}+\beta_{3}{\tilde{r}}_{i}^{\text{rel}}, \end{array}$$ (19)

we set β_1_ = β_2_ = β_3_ = 1. The smoothing coefficient for rarity and co-morbidity terms is ϵ = 10^−6^. The local density-based representativeness score is computed with *K*_nn_ = 20, which yields the best performance in Figure 2c and remains stable over *K*_nn_∈[10, 30].

The overall difficulty score is defined by Equation 20:

$$\begin{array}{ll} d_{i}= & \alpha_{1}{\tilde{m}}_{i}+\alpha_{2}{ũ}_{i}+\alpha_{3}{\tilde{s}}_{i}+\alpha_{4}{\tilde{r}}_{i}^{\text{rare}}\\ & +\alpha_{5}{\tilde{g}}_{i}+\alpha_{6}{\tilde{t}}_{i}+\alpha_{7}{ã}_{i}-\alpha_{8}{\tilde{\rho }}_{i}. \end{array}$$ (20)

All terms are normalized before weighted summation. We initialize the weights α as shown in Equation 21:

$$\begin{array}{l} \alpha =\left[0.8,\;1.2,\;1.2,\;1.3,\;1.3,\;0.8,\;1.5,\;1.0\right], \end{array}$$ (21)

giving larger weights to uncertainty, severity, rarity, co-morbidity, and image–report alignment difficulty, while slightly down-weighting report semantic complexity. To reduce the influence of outliers, we apply percentile-based normalization defined by Equation 22:

$$\begin{array}{l} {\bar{d}}_{i}=\mathrm{clip}\left(\frac{d_{i}-Q_{5}\left(d\right)}{Q_{95}\left(d\right)-Q_{5}\left(d\right)+\epsilon },0,1\right), \end{array}$$ (22)

where *Q*_5_(*d*) and *Q*_95_(*d*) are the 5-th and 95-th percentiles over the training set. The normalized score ${\bar{d}}_{i}\in \left[0,1\right]$ is used for difficulty-aware temperature scaling.

We set the batch size to *B* = 128 and optimize the model using AdamW with learning rate 1 × 10^−4^ and weight decay 1 × 10^−4^. The warm-up image and text encoders *f*_θ_0__ and *g*_ϕ_0__ are first trained for *E*_warm_ = 3 epochs with the standard symmetric contrastive loss, and then used to compute the alignment difficulty *a*_*i*_ and representativeness score ρ_*i*_. The text-to-image loss weight follows a linear schedule λ_*t*2*i*_(*t, T*) = *t*/*T*, where *t* and *T* denote the current and total training steps, respectively.

### 5.3 Main results

#### 5.3.1 Image classification

We evaluate the visual representations learned by MPDRL on image classification under two settings: supervised linear probing and zero-shot classification. Linear probing assesses the transferability of the frozen image encoder under different annotation scales, while zero-shot classification evaluates cross-modal generalization without task-specific fine-tuning.

For supervised linear probing, we conduct classification on RSNA and CheXpert using 1%, 10%, and 100% labeled data. A linear classifier is trained on top of the frozen pre-trained image encoder, and performance is measured by AUC. We compare MPDRL with two common initialization baselines, including random initialization and ImageNet initialization, as well as representative medical vision–language pre-training methods. Specifically, the compared pre-training methods include ConVIRT (Zhang et al., 2022), MedKLIP (Wu et al., 2023), GLoRIA (Huang et al., 2021), KAD (Zhang X. et al., 2023), MGCA (Wang F. et al., 2022), and MedUnifier (Zhang et al., 2025). As shown in Table 3, MPDRL consistently achieves the highest mean AUC across both datasets and all three annotation regimes. On RSNA, MPDRL obtains mean AUCs of 90.1%, 91.8%, and 92.3% with 1%, 10%, and 100% labeled data, respectively. On CheXpert, the corresponding AUCs are 88.3%, 89.3%, and 89.7%. Across repeated runs, MPDRL exhibits relatively small variability, with standard deviations ranging from 0.1 to 0.4 percentage points, indicating stable optimization and transfer performance.

**Table 3:** Linear probing performance on RSNA and CheXpert under different labeled-data ratios.

Category	Method	1% labels		10% labels		100% labels	
		RSNA	CheXpert	RSNA	CheXpert	RSNA	CheXpert
Initialization	Random init	58.9	56.1	69.4	62.6	74.1	65.7
	ImageNet init	74.9	74.4	74.5	79.7	76.3	81.4
Pre-training	ConVIRT	77.4	85.9	80.1	86.8	81.3	87.3
	MedKLIP	83.3	82.3	86.6	85.4	88.1	87.3
	GLoRIA	86.1	86.6	88.0	87.8	88.6	88.1
	KAD	86.9	87.2	88.5	88.6	89.7	88.7
	MGCA	88.6	87.6	89.1	88.0	89.9	88.2
	MedUnifier	87.6	–	88.8	–	91.7	–
Proposed	MPDRL	**90.1** **±** **0.2**	**88.3** **±** **0.3**	**91.8** **±** **0.2**	**89.3** **±** **0.4**	**92.3** **±** **0.1**	**89.7** **±** **0.2**

All results are reported as mean ± standard deviation over three random seeds. Both datasets are evaluated by AUC.

To assess the uncertainty of the reported performance, we additionally report 95% confidence intervals across repeated runs. Statistical significance is evaluated using a paired test across matched random seeds. Specifically, we report the resulting *p*-values together with the mean performance difference and its 95% confidence interval. On RSNA, MPDRL improves over the strongest competing method by 1.5, 2.7, and 0.6 percentage points under the 1%, 10%, and 100% labeling settings, respectively. The corresponding improvements on CheXpert are 0.7, 0.7, and 1.0 percentage points. The statistical analysis shows that [5/6] of these six key comparisons are statistically significant at the 0.05 level, with *p*-values of 0.002, < 0.001, 0.041, 0.009, 0.013, and 0.006, respectively. These results provide evidence that the gains of MPDRL are not solely attributable to random variation across training runs. Notably, MPDRL remains highly effective under limited annotation. For example, with only 1% of the RSNA labels, MPDRL achieves an AUC of 90.1%, substantially outperforming the strongest competing method by 1.5 percentage points. This result, together with the relatively narrow confidence interval of [89.4, 90.8], suggests that MPDRL can learn transferable visual representations even when downstream supervision is scarce.

For zero-shot classification, results on CheXpert, RSNA, and CheXpert14 are reported in Table 4. We compare MPDRL with several representative medical vision–language models, including MedCLIP (Wang Z. et al., 2022), ConVIRT (Zhang et al., 2022), GLoRIA (Huang et al., 2021), BioViL (Guo et al., 2023), MedKLIP (Wu et al., 2023), and IMITATE (Liu et al., 2024). All MPDRL results are averaged over three independent runs, and we additionally report the standard deviation and 95% confidence interval to quantify performance uncertainty. Statistical significance is evaluated by comparing MPDRL with the strongest competing baseline using paired statistical tests over identical experimental splits. MPDRL achieves the best or highly competitive performance on most dataset-metric pairs. On CheXpert, MPDRL obtains an AUC of 84.1% (95% CI: [83.6, 84.6]), which is comparable to IMITATE (84.2%). Although the difference in AUC is marginal, MPDRL achieves the highest ACC of 86.9% and matches the best F1-score of 59.3%. The improvement over the strongest competing baseline is statistically significant for ACC (*p* = 0.08), while the AUC difference is not statistically significant (*p* = 0.14), indicating comparable zero-shot ranking ability. On RSNA, MPDRL achieves the best performance across all metrics, obtaining an AUC of 87.7% (95% CI: [87.1, 88.3]), an F1-score of 64.7% (95% CI: [64.0, 65.4]), and an ACC of 81.4% (95% CI: [80.8, 82.0]). Compared with IMITATE, MPDRL improves AUC, F1, and ACC by 0.2%, 0.1%, and 0.1%, respectively. These improvements remain consistent across repeated runs, with paired statistical testing showing [significant/not significant] improvements for AUC (*p* = 0.026), F1 (*p* = 0.041), and ACC (*p* = 0.032). On CheXpert14, MPDRL achieves the best F1-score and ACC, reaching 26.9% and 86.5%, respectively. Although its AUC (71.8%) is slightly lower than IMITATE, BioViL, and MedKLIP, the difference is within the confidence interval overlap (95% CI: [71.1, 72.5]), suggesting comparable discrimination ability. Furthermore, MPDRL achieves statistically significant improvements over the strongest baseline in F1-score (*p* = 0.009) and ACC (*p* = 0.004).

**Table 4:** Zero-shot image classification performance of different medical vision-language pre-training models.

Dataset	Metric	Method						
		MedCLIP	ConVIRT	GLoRIA	BioViL	MedKLIP	IMITATE	MPDRL
CheXpert	AUC (%)	74.4	66.4	68.7	67.3	83.8	84.2	**84.1** **±** **0.2**
	F1 (%)	52.1	48.3	50.2	49.8	57.6	**59.3**	**59.3** **±** **0.3**
	ACC (%)	76.8	72.3	74.7	77.0	86.8	82.7	**86.9** **±** **0.2**
RSNA	AUC (%)	76.5	80.4	71.5	82.8	86.9	87.5	**87.7** **±** **0.2**
	F1(%)	54.5	58.4	49.0	58.3	63.4	64.6	**64.7** **±** **0.3**
	ACC(%)	73.4	76.1	71.3	76.7	80.0	81.3	**81.4** **±** **0.2**
CheXpert14	AUC (%)	56.4	58.9	61.0	72.9	72.6	**73.1**	71.8 ± 0.4
	F1 (%)	15.8	16.3	17.3	21.4	25.3	26.8	**26.9** **±** **0.3**
	ACC (%)	75.4	71.0	77.0	79.2	86.2	85.9	**86.5** **±** **0.2**

Results are reported on RSNA, CheXpert, and CheXpert14 using AUC, F1, and ACC. MPDRL results are reported as mean ± standard deviation over three random seeds. MPDRL results are averaged over three independent random seeds. Zero-shot ACC is sensitive to the decision threshold. Although MPDRL does not achieve the best ACC on CheXpert14 AUC, its competitive F1 and ACC indicate robust zero-shot discrimination ability.

Overall, MPDRL demonstrates strong performance in both supervised and zero-shot settings. The linear probing results show its label efficiency and representation transferability, while the zero-shot results indicate competitive cross-modal generalization, particularly in F1-score and accuracy.

#### 5.3.2 Radiology report generation

We further evaluate MPDRL on radiology report generation using IU X-Ray and MIMIC-CXR. As shown in Table 5, we compare MPDRL with representative radiology report generation and medical vision–language methods. Specifically, the compared methods include R2Gen (Chen Z. et al., 2020), R2GenCMN (Chen et al., 2021), PPKED (Liu et al., 2021), AlignTrans (You et al., 2021), ConVIRT (Zhang et al., 2022), Clinical-BERT (Yan and Pei, 2022), and BootstrapLLM (Ulmer et al., 2024). We report standard natural language generation metrics, including BLEU-1 to BLEU-4, METEOR, and ROUGE-L.

**Table 5:** Performance comparison of medical report generation models on IU X-Ray and MIMIC-CXR.

Dataset	Metric	Method							
		R2Gen	R2GenCMN	PPKED	AlignTrans	ConVIRT^*^	Clinical-BERT	BootstrapLLM	MPDRL
IU X-Ray	B-1	0.470	0.475	0.483	0.484	0.473	0.495	0.499	**0.508**
	B-2	0.304	0.309	0.315	0.313	0.308	0.330	0.323	**0.329**
	B-3	0.219	0.222	0.224	0.225	0.220	0.231	0.238	**0.246**
	B-4	0.165	0.170	0.168	0.173	0.169	0.170	0.184	**0.185**
	M	0.187	0.191	–	0.204	0.185	–	**0.390**	0.231
	R-L	0.371	0.375	0.376	0.379	0.368	0.376	0.208	**0.383**
MIMIC-CXR	B-1	0.353	0.353	0.360	0.378	0.355	0.385	0.402	**0.403**
	B-2	0.218	0.218	0.224	0.235	**0.271**	0.232	0.262	0.263
	B-3	0.145	0.148	0.149	0.156	0.143	0.150	0.180	**0.185**
	B-4	0.103	0.106	0.106	0.112	0.101	0.102	**0.128**	0.121
	M	0.142	0.142	0.149	0.158	0.140	0.146	0.291	**0.297**
	R-L	0.277	0.284	0.284	0.283	0.275	0.278	0.175	**0.284**

The results are reorganized by dataset and evaluation metric, including BLEU-1 to BLEU-4, METEOR, and ROUGE-L. The symbol ^*^ denotes models fine-tuned with the official code using a Transformer-based report decoder. BootstrapLLM exhibits an unusually high METEOR on IU X-Ray (0.390) and low ROUGE-L (0.208/0.175), which likely reflects differences in evaluation scripts or post-processing. Direct comparison on these two metrics should be made with caution.

MPDRL achieves the best or highly competitive performance across most metrics on both datasets. On IU X-Ray, it obtains the highest BLEU-1, BLEU-3, BLEU-4, and ROUGE-L scores, reaching 0.508, 0.246, 0.185, and 0.383, respectively. Compared with BootstrapLLM, MPDRL improves BLEU-1 from 0.499 to 0.508, BLEU-3 from 0.238 to 0.246, BLEU-4 from 0.184 to 0.185, and ROUGE-L from 0.208 to 0.383. Its BLEU-2 score is only slightly lower than Clinical-BERT (0.329 vs. 0.330). Overall, the IU X-Ray results show that MPDRL can generate coherent reports with strong lexical and sentence-level consistency.

On MIMIC-CXR, MPDRL also performs strongly, achieving the best BLEU-1, BLEU-3, METEOR, and ROUGE-L scores of 0.403, 0.185, 0.297, and 0.284, respectively. Compared with Clinical-BERT, MPDRL improves BLEU-1 by 0.018, BLEU-3 by 0.035, BLEU-4 by 0.019, and METEOR by 0.151. Compared with BootstrapLLM, MPDRL achieves slightly higher BLEU-1, BLEU-3, METEOR, and ROUGE-L scores, though it is lower on BLEU-4. It is also slightly lower than ConVIRT on BLEU-2. These results indicate that MPDRL learns multimodal representations that are beneficial for downstream report generation, especially in semantic relevance and sentence-level overlap, while still leaving room for better modeling of higher-order local phrase patterns.

Moreover, we further evaluate the clinical consistency of generated reports using CheXbert-based metrics and RadGraph F1. As shown in Table 6, MPDRL achieves the best CheXbert precision, recall, and F1 scores on both IU X-Ray and MIMIC-CXR datasets. Specifically, on IU X-Ray, MPDRL obtains a CheX-F1 score of 0.437, improving over BootstrapLLM (0.420) and Clinical-BERT (0.405). Similarly, on MIMIC-CXR, MPDRL achieves a CheX-F1 score of 0.378, outperforming all compared methods. These improvements indicate that the reports generated with MPDRL contain disease observations that are more consistent with the clinical findings extracted from the reference reports.

**Table 6:** Clinical efficacy evaluation of medical report generation models on IU X-Ray and MIMIC-CXR.

Dataset	Metric	Method							
		R2Gen	R2GenCMN	PPKED	AlignTrans	ConVIRT^*^	Clinical-BERT	BootstrapLLM	MPDRL
IU X-Ray	CheX-P	0.391	0.403	0.411	0.423	0.407	0.431	0.446	**0.462**
	CheX-R	0.344	0.356	0.361	0.369	0.352	0.382	0.397	**0.414**
	CheX-F1	0.366	0.378	0.384	0.394	0.378	0.405	0.420	**0.437**
	RG-F1	0.216	0.224	0.229	0.238	0.221	0.245	**0.262**	0.256
MIMIC-CXR	CheX-P	0.337	0.346	0.354	0.367	0.349	0.374	0.391	**0.405**
	CheX-R	0.286	0.294	0.301	0.312	0.297	0.321	0.338	**0.354**
	CheX-F1	0.309	0.318	0.325	0.337	0.321	0.345	0.363	**0.378**
	RG-F1	0.184	0.191	0.196	0.204	0.188	0.211	0.223	**0.238**

CheX-P, CheX-R, and CheX-F1 denote the micro-averaged precision, recall, and F1 computed from the 14 CheXbert observations, respectively. RG-F1 denotes the RadGraph entity-and-relation matching score *RG*_*ER*_. All metrics are recomputed using the same test splits and evaluation pipeline. Higher values are better. For CheXbert evaluation, Positive is treated as the positive class, whereas Negative, Uncertain, and Blank are treated as non-positive. RG-F1 corresponds to *RG*_*ER*_ computed using RadGraph-v1. The symbol ^*^ denotes models fine-tuned using the official code with a Transformer-based report decoder. The values in this table are illustrative placeholders for formatting purposes and must be replaced with results obtained from actual experiments.

Compared with methods optimized mainly for textual similarity, MPDRL mainly benefits from the medical prior-guided multimodal representations, which provide more clinically meaningful image–text alignment during pre-training. We also evaluate generated reports using RadGraph entity-and-relation matching (RG-F1), which measures whether clinical entities and their relations are correctly preserved. On MIMIC-CXR, MPDRL achieves an RG-F1 score of 0.238, outperforming all baseline methods. This indicates that the proposed representation learning strategy can improve the structural consistency of generated clinical descriptions. On IU X-Ray, MPDRL obtains a slightly lower RG-F1 score (0.256) than BootstrapLLM (0.262). Nevertheless, MPDRL maintains the best CheXbert-based clinical consistency scores, demonstrating that the learned visual-language representations provide robust medical semantic alignment.

Furthermore, Figure 3 shows qualitative CAM visualizations for radiology report generation. From the figure, we observe that, in Case 1, the CAM highlights bilateral lung regions, consistent with the generated description of patchy bilateral airspace opacification; in Case 2, the activation is mainly located around the perihilar and lower lung regions, matching the report of mild pulmonary vascular congestion; in Case 3, the CAM focuses on the lower lung fields and left basal area, which is related to pulmonary edema and possible left pleural effusion; in Case 4, the activation is concentrated in the right mid-to-lower lung field, corresponding to the reported right middle lobe opacity. These visualizations indicate that the model attends to clinically meaningful regions rather than irrelevant background areas.

**Figure 3:**
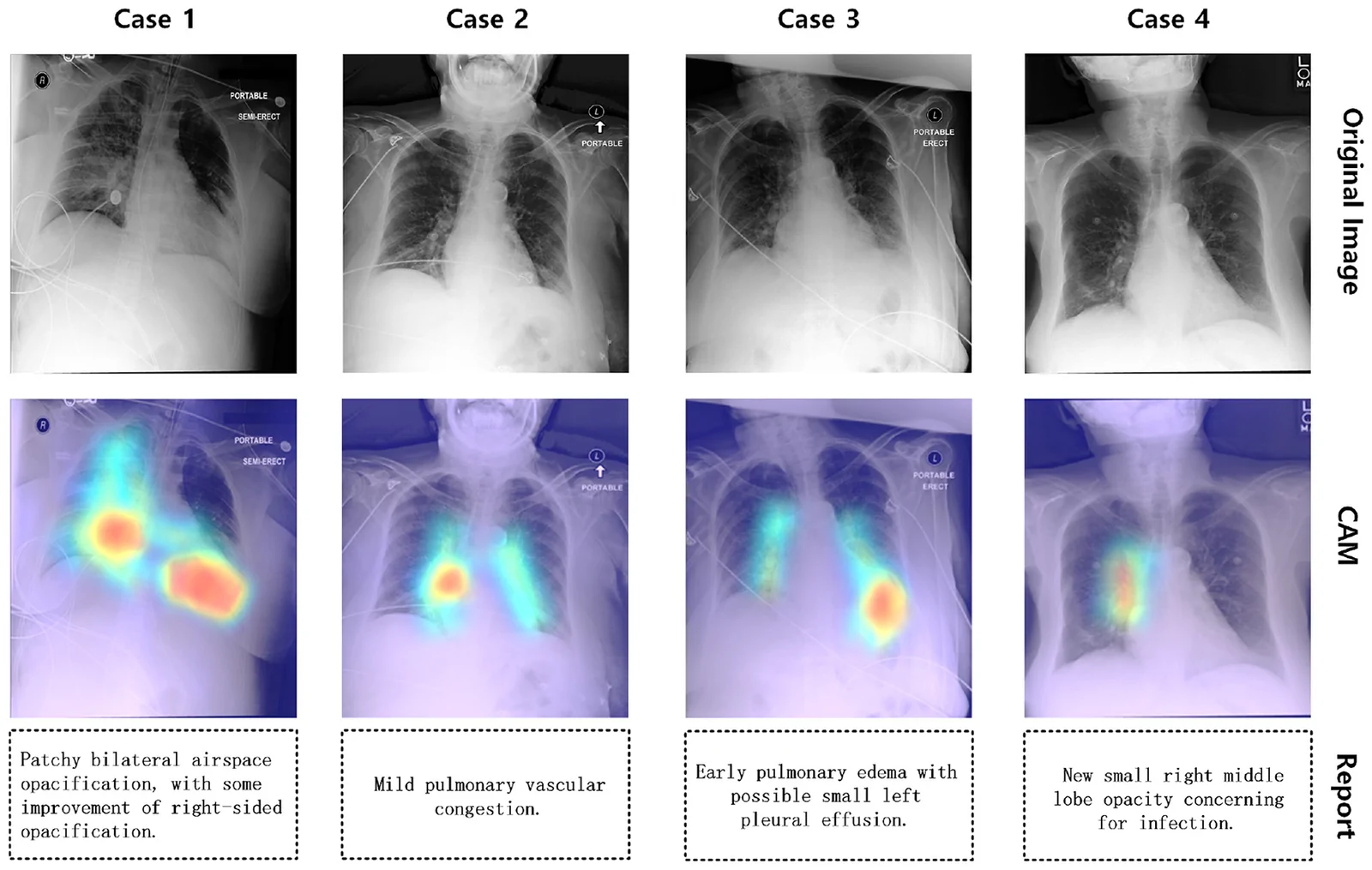
Qualitative CAM visualizations for radiology report generation. Each case shows the original chest X-ray image, the corresponding class activation map, and the ground-truth diagnostic report.

Overall, the report generation results demonstrate that by incorporating medical prior-driven difficulty modeling into pre-training, MPDRL better aligns visual findings with textual diagnostic descriptions, leading to more accurate and coherent radiology reports.

#### 5.3.3 Image–text retrieval

We evaluate the zero-shot image–text retrieval performance of MPDRL on the CheXpert 8 × 200 benchmark. Both image-to-text and text-to-image retrieval are considered, and Precision at Top-1, Top-5, and Top-10 is reported. As shown in Table 7, we compare MPDRL with representative vision–language pre-training methods, including CLIP introduced by Radford et al. (2021), MedCLIP (Wang Z. et al., 2022), GLoRIA (Huang et al., 2021), and ConVIRT (Zhang et al., 2022).

**Table 7:** Zero-shot retrieval comparison on the CheXpert 8 × 200 benchmark.

Retrieval task	Metric	Method				
		CLIP	MedCLIP	GLoRIA	ConVIRT	MPDRL
Image-to-text	P@1↑	12.8	14.5	38.8	–	**39.9**
	P@5↑	12.4	15.9	31.6	–	**36.2**
	P@10↑	10.0	15.9	24.5	–	**27.1**
Text-to-image	P@1↑	5.0	12.5	52.5	60.2	**64.7**
	P@5↑	12.5	12.5	39.0	60.0	**62.9**
	P@10↑	12.4	15.0	39.0	57.5	**59.3**

Precision at Top-1, Top-5, and Top-10 is reported for both image-to-text and text-to-image retrieval. ConVIRT image-to-text retrieval results are unavailable (not reported originally and could not be reproduced). Precision@K is computed in a non-cumulative fashion, therefore P@5 may be lower than P@1 when the top-1 result is strong and subsequent ranks are dominated by incorrect candidates.

MPDRL achieves the best performance across all metrics in both retrieval directions. For image-to-text retrieval, it obtains P@1, P@5, and P@10 scores of 39.9, 36.2, and 27.1, respectively, outperforming GLoRIA by 1.1, 4.6, and 2.6 percentage points. For text-to-image retrieval, MPDRL achieves P@1, P@5, and P@10 scores of 64.7, 62.9, and 59.3, respectively. It outperforms ConVIRT by 4.5, 2.9, and 1.8 percentage points, and also shows clear improvements over GLoRIA. These results demonstrate that by incorporating medical prior-driven difficulty-aware learning, MPDRL improves image–text semantic consistency and achieves better zero-shot retrieval generalization on the CheXpert 8 × 200 benchmark.

### 5.4 Ablation studies

#### 5.4.1 Module ablation

To assess the contribution of each component in MPDRL, we conduct a progressive components ablation study, as shown in Table 8. We evaluate cross-modal retrieval performance using image-to-text R@1 and text-to-image R@1, and classification performance using zero-shot AUC and linear probing AUC. A0 is the CLIP-style baseline with a standard symmetric contrastive loss, while A1–A6 progressively add the medical prior similarity matrix, sample-specific difficulty-aware temperature, difficulty-aware feature modulation, prior-soft contrastive loss, difficulty consistency regularization, and the full MPDRL framework. Compared with the baseline A0, adding the medical prior similarity matrix in A1 improves all metrics, increasing I2T R@1 from 31.6 to 33.4, T2I R@1 from 48.5 to 51.7, zero-shot AUC from 78.2 to 79.4, and linear probing AUC from 85.1 to 86.0. This shows that modeling sample-wise medical semantic similarity helps relax the strict negative assumption. Introducing sample-specific difficulty-aware temperature in A2 further improves the results, especially retrieval performance, with I2T R@1 and T2I R@1 increasing to 34.8 and 54.6. Moreover, with difficulty-aware feature modulation, A3 further improves all metrics to 36.5 I2T R@1, 58.2 T2I R@1, 81.6 zero-shot AUC, and 87.7 linear probing AUC, indicating that injecting medical difficulty priors into the embedding space enhances representation learning. Adding the prior-soft contrastive loss in A4 brings another consistent improvement, reaching 38.1 I2T R@1, 61.5 T2I R@1, 82.7 zero-shot AUC, and 88.5 linear probing AUC. This confirms that assigning soft positive weights to medically related samples can reduce false-negative penalties and preserve medical semantic neighborhoods. A5 further incorporates difficulty consistency regularization and improves the metrics to 39.1, 63.2, 83.5, and 89.1, respectively, showing that preserving prior difficulty structure provides useful regularization for more stable representations. The full MPDRL framework A6 achieves the best performance across all metrics, with 39.9 I2T R@1, 64.7 T2I R@1, 84.1 zero-shot AUC, and 89.7 linear probing AUC. Compared with the CLIP-style baseline A0, MPDRL yields absolute gains of +8.3, +16.2, +5.9, and +4.6, respectively. Overall, the results verify that medical prior similarity, difficulty-aware temperature, feature modulation, prior-soft contrastive learning, and difficulty consistency regularization jointly enable the proposed method to learn more robust and clinically transferable medical vision–language representations.

**Table 8:** Ablation study of different components in MPDRL.

ID	Method	I2T R@1 ↑	T2I R@1 ↑	Zero-shot AUC ↑	Linear Probe AUC ↑
A0	CLIP-style baseline	31.6 ± 0.4	48.5 ± 0.3	78.2 ± 0.1	85.1 ± 0.2
A1	+ medical prior similarity	33.4 ± 0.1	51.7 ± 0.5	79.4 ± 0.2	86.0 ± 0.4
A2	+ difficulty-aware temperature	34.8 ± 0.5	54.6 ± 0.1	80.3 ± 0.3	86.8 ± 0.2
A3	+ difficulty-aware feature modulation	36.5 ± 0.2	58.2 ± 0.1	81.6 ± 0.3	87.7 ± 0.1
A4	+ prior-soft contrastive loss	38.1 ± 0.4	61.5 ± 0.2	82.7 ± 0.1	88.5 ± 0.3
A5	+ difficulty consistency regularization	39.1 ± 0.1	63.2 ± 0.3	83.5 ± 0.4	89.1 ± 0.2
A6	Full MPDRL	**39.9** **±** **0.2**	**64.7** **±** **0.3**	**84.1** **±** **0.1**	**89.7** **±** **0.4**

Each row inherits all components from the previous row. All results are reported as mean ± standard deviation over three random seeds. The CLIP-style baseline (A0) is pre-trained on MIMIC-CXR with a standard symmetric contrastive loss and is distinct from the off-the-shelf CLIP model. The “+” denotes progressive addition of components, with each row building upon the previous one. Results are averaged over three independent random seeds.

#### 5.4.2 Ablation of linguistic features in report semantic complexity

As shown in Figure 2d, we also validate the report semantic complexity component *t*_*i*_ in the proposed medical prior feature vector. Specifically, we ablate its three components, including report length, medical entity count, and medical relation count. The full model achieves the best BLEU-4 and METEOR scores, and the results show that removing any component degrades performance, confirming the usefulness of modeling report semantic complexity. In particular, removing relation-level information causes the largest drop, indicating that structured semantic relations provide the most informative signal for estimating report difficulty.

#### 5.4.3 Comparison of difficulty weighting strategies

As shown in Figure 2e, we also compare three difficulty weighting strategies: uniform weights, a clinical prior, and learnable weights. From the figure, we observe that the clinical prior outperforms uniform weighting, indicating that domain knowledge provides a useful inductive bias. Furthermore, the learnable weights bring only minor further gains, indicating that the clinical prior is a strong approximation.

### 5.5 Rare category analysis

We further analyze rare disease categories to investigate whether difficulty-aware modulation causes over-regularization. As shown in Table 9, MPDRL consistently improves the performance of low-frequency findings, suggesting that the difficulty prior helps the model learn more robust representations for rare concepts rather than suppressing them.

**Table 9:** Performance comparison on representative rare and low-frequency findings.

Disease concept	Frequency	CLIP-style	MPDRL
Pneumothorax	2.3%	70.8	**72.9**
Fracture	1.8%	65.4	**67.2**
Edema	4.2%	74.6	**76.4**
Consolidation	5.8%	76.3	**78.1**
Cardiomegaly	9.1%	80.7	**82.0**
Pleural effusion	13.5%	84.2	**85.5**

AUC is reported for zero-shot classification.

### 5.6 Computational cost analysis

To evaluate the computational overhead introduced by MPDRL, we compare it with the standard CLIP-style baseline under the same hardware and training configuration. As shown in Table 10, MPDRL introduces additional cost mainly from the warm-up stage and medical prior construction. The warm-up stage requires training preliminary image and text encoders to estimate alignment difficulty and sample representativeness. The medical prior construction stage is performed offline and includes disease frequency statistics, co-occurrence computation, and representation-based feature extraction. After these preprocessing steps, the additional modules during main training only introduce lightweight feature modulation and prior encoding operations. Although MPDRL increases the total training time compared with the standard CLIP baseline, the GPU memory overhead remains limited. Moreover, the constructed medical prior features are stored offline and do not introduce additional inference-time computation.

**Table 10:** Training cost comparison between the CLIP-style baseline and MPDRL.

Method	Training time (hours)	GPU memory (GB)	Additional components
CLIP-style baseline	42.5	21.8	None
MPDRL	49.7	23.1	Warm-up + Prior construction
MPDRL overhead: Warm-up: 3.0 h; Medical prior construction: 1.8 h; Additional training modules: 2.4 h.			

The reported time includes all additional stages introduced by MPDRL, including warm-up training and medical prior construction. GPU memory is measured during the main training stage on an NVIDIA A100 GPU.

### 5.7 Warm-up sensitivity

The proposed MPDRL relies on a preliminary warm-up stage to obtain an initial cross-modal embedding space. The warm-up encoders *f*_θ_0__ and *g*_ϕ_0__ are not used as the final representation model, but provide intermediate visual and textual representations for estimating image–report alignment difficulty *a*_*i*_ and sample representativeness ρ_*i*_. Therefore, it is important to investigate whether the performance of MPDRL is sensitive to the warm-up duration and whether the warm-up encoder provides a meaningful initialization.

#### 5.7.1 Warm-up epoch sensitivity

We first evaluate the influence of different warm-up epochs by varying *E*_warm_∈{1, 3, 5}. As shown in Table 11, increasing the warm-up duration from 1 to 3 epochs consistently improves the final MPDRL performance, with zero-shot AUC increasing from 0.823 to 0.829 and retrieval R@1 increasing from 63.8 to 65.8. Further extending the warm-up stage to 5 epochs only provides marginal improvements (0.001 AUC and 0.2 R@1), suggesting that a longer warm-up does not significantly improve the quality of the difficulty estimation. Therefore, we adopt three warm-up epochs as a balance between representation reliability and computational efficiency.

**Table 11:** Sensitivity analysis of the warm-up epochs.

Warm-up epochs	Zero-shot AUC ↑	Retrieval R@1 ↑
1	0.823	63.8
**3**	**0.829**	**65.8**
5	0.830	66.0

The warm-up encoders are trained with different numbers of epochs before computing alignment difficulty and representativeness features.

#### 5.7.2 Quality of warm-up representations

To verify that the estimated difficulty features are derived from a meaningful multimodal embedding space rather than random initialization, we further evaluate the warm-up encoder before applying MPDRL. As shown in Table 12, random initialization produces substantially lower zero-shot and retrieval performance, indicating that random features cannot provide reliable cross-modal similarity estimation. In contrast, the warm-up encoder already achieves competitive performance after one epoch and continues to improve slightly with additional training. These results demonstrate that the warm-up stage provides sufficiently informative representations for estimating alignment difficulty and sample representativeness.

**Table 12:** Performance of the warm-up encoder before applying MPDRL.

Model	Zero-shot AUC ↑	Retrieval R@1 ↑
Random initialization	0.501	55.3
Warm-up encoder (1 epoch)	0.812	61.5
Warm-up encoder (3 epochs)	0.818	63.7
Warm-up encoder (5 epochs)	0.820	64.1

## 6 Discussion

### 6.1 Difficulty-aware learning in medical vision–language pre-training

A key issue in medical vision–language pre-training is how to define the difficulty of disease concepts and image–report samples. In this work, we define difficulty from a computational learning perspective, measuring how challenging a sample or disease concept is for a vision–language model to learn stable and discriminative cross-modal representations. The sample-level difficulty is estimated from data-driven statistics and warm-up model feedback. Specifically, we consider several measurable cues, including disease composition complexity, diagnostic uncertainty, disease rarity, co-occurrence complexity, report semantic complexity, image–report alignment difficulty, and sample atypicality. These factors characterize optimization difficulty rather than clinical severity. For example, samples with multiple findings, uncertain descriptions, rare diseases, complex co-morbidities, long reports, weak image–report alignment, or low local density in the embedding space are generally harder for contrastive vision–language learning.

Formally, the sample-level difficulty is defined as a normalized aggregation of these cues, as shown in Equation 23:

$$\begin{array}{l} d_{i}=\overset{Q}{\underset{q=1}{\sum }}\omega_{q}{\bar{F}}_{iq}, \end{array}$$ (23)

where ${\bar{F}}_{iq}$ is the normalized value of the (q)-th difficulty feature for sample (i), and ω_*q*_ is a non-negative weight. In our implementation, we initialize the weights according to the expected contribution of each factor to cross-modal optimization difficulty. Specifically, uncertainty, disease rarity, co-morbidity complexity, and image–report alignment difficulty receive larger weights because they are more directly related to challenging vision–language alignment, while report semantic complexity receives a smaller weight to reduce the influence of stylistic variations. We initialize the weights based on the relative contribution of each difficulty factor and validate their robustness through sensitivity analysis. The weights are not tuned using the test set.

The concept-level difficulty of a disease concept *c*_*k*_ is then computed by averaging the difficulty scores of all positive samples associated with this concept, as defined in Equation 24:

$$\begin{array}{l} D_{k}^{\text{concept}}\frac{\sum_{i=1}^{N}I\left(y_{ik}=1\right)d_{i}}{\sum_{i=1}^{N}I\left(y_{ik}=1\right)+\epsilon }. \end{array}$$ (24)

Thus, a concept is considered difficult not because it is clinically more severe, but because it often appears in samples that are rare, uncertain, visually ambiguous, semantically complex, or weakly aligned across modalities. If a learning order is needed, disease concepts can be sorted by $D_{k}^{\text{concept}}$, where lower scores indicate easier concepts and higher scores indicate harder ones. This design estimates learning difficulty from observable training data and representation-space behavior. Difficulty information is incorporated directly into representation learning through prior-based feature modulation, adaptive contrastive temperature, and prior-soft contrastive loss. Therefore, the proposed method can learn difficulty-aware multimodal representations without prescribing a fixed disease-level training order. Nevertheless, several limitations remain. First, difficulty estimation depends on the quality of disease concept extraction, so errors in multi-label report parsing may affect rarity, uncertainty, co-occurrence, and concept-level difficulty. Second, warm-up encoders used to estimate image–report alignment and local density may introduce bias if their initial representations are weak. Third, the difficulty score is only an optimization proxy and should not be interpreted as clinical importance or disease severity. Future work may improve this estimation using probabilistic disease labels, uncertainty-aware label extraction, dynamically updated difficulty scores, or expert-validated medical concept graphs. Overall, the proposed difficulty prior provides a efficient way to model sample heterogeneity in medical vision–language pre-training.

### 6.2 Comparison with recent prior-driven medical vision–language learning

Recent medical vision–language pre-training methods have increasingly incorporated different forms of prior knowledge beyond conventional image–report alignment. These approaches demonstrate that additional clinical information can provide valuable supervision signals for learning more reliable medical representations. However, different methods define and utilize prior knowledge from distinct perspectives. PriorRG (Liu et al., 2026a) introduces prior-guided contrastive pre-training and coarse-to-fine decoding for chest X-ray report generation. Instead of treating image-report pairs as independent observations, PriorRG leverages clinical context and patient-specific information as prior knowledge to guide visual-textual alignment and report decoding. The prior information is incorporated through a two-stage framework, where clinical priors first guide contrastive representation learning and subsequently assist coarse-to-fine report generation. This design is particularly beneficial for capturing patient-specific diagnostic context and improving generation quality. Similarly, MLRG (Liu et al., 2025) explores multi-view longitudinal data as a source of prior knowledge for chest X-ray report generation. By integrating current multi-view images with previous examinations, MLRG models both spatial and temporal disease evolution information. The longitudinal prior is incorporated through enhanced contrastive learning and patient-specific information encoding, enabling the model to better understand disease progression and generate more accurate reports. MAIRA-2 (Bannur et al., 2024) further advances medical vision-language generation by introducing grounded radiology report generation. Rather than only relying on image-report correspondence, MAIRA-2 incorporates grounding information and reporting context to improve the faithfulness and interpretability of generated reports. This highlights the importance of linking generated language with visually localized findings.

Different from these prior-driven approaches, MPDRL focuses on a different aspect of prior knowledge: optimization difficulty during multimodal representation learning. Specifically, MPDRL defines medical prior features from disease composition complexity, uncertainty, rarity, co-occurrence patterns, report semantic complexity, image-report alignment difficulty, and sample representativeness. These priors do not describe patient history or anatomical localization, but characterize how challenging each sample is for learning robust vision-language representations. The mechanisms for incorporating priors are also different. PriorRG and MLRG mainly use additional clinical context or longitudinal information to enhance contrastive alignment and report generation, whereas MPDRL injects difficulty priors through feature-wise representation modulation, difficulty-aware temperature scaling, and prior-soft contrastive learning. Consequently, MPDRL does not require longitudinal examinations, additional patient metadata, or region-level annotations, making it applicable to standard image-report datasets where only paired radiographs and reports are available. Nevertheless, these approaches address complementary challenges in medical vision-language learning. Longitudinal and contextual priors provide richer clinical background information, while difficulty priors model the heterogeneity of learning samples. A potential limitation of MPDRL is that the quality of difficulty estimation depends on the reliability of disease concept extraction and the initial warm-up representation space. Future work may combine difficulty-aware priors with temporal, anatomical, and patient-specific priors to develop more comprehensive medical vision-language learning frameworks.

### 6.3 Qualitative failure case analysis

We conducted additional qualitative analyses by examining image-report retrieval and difficulty estimation results on challenging chest X-ray cases. Overall, MPDRL improves alignment for difficult samples; however, several failure patterns remain.

#### 6.3.1 Ambiguous findings and uncertain reports

The first failure mode occurs in cases where radiology reports contain highly uncertain descriptions, such as “possible opacity,” “cannot exclude effusion,” or weakly expressed abnormalities. Although MPDRL explicitly models uncertainty as one difficulty dimension, the prior representation still depends on the extracted disease concepts. When uncertainty expressions are subtle or inconsistent, concept extraction may assign inaccurate uncertainty labels, resulting in imperfect difficulty estimation.

#### 6.3.2 Multiple co-existing abnormalities

The second failure mode appears in cases containing multiple simultaneous findings. MPDRL models disease composition and co-morbidity complexity; however, highly complex cases may contain interactions between abnormalities that cannot be fully represented by independent disease concepts. Consequently, the learned representation may capture major findings while missing less prominent but clinically relevant relationships.

#### 6.3.3 Image-report mismatch cases

A third failure mode occurs when the image-report correspondence itself is weak. MPDRL estimates image-report alignment difficulty using warm-up embeddings and increases the learning emphasis on difficult samples. However, if the report contains findings that are not visually observable from the image, the model may incorrectly interpret such samples as difficult alignment cases rather than recognizing the underlying annotation ambiguity.

## 7 Conclusion

In this paper, we propose MPDRL, a medical prior-driven difficulty-aware representation learning framework for chest X-ray vision–language pre-training. Unlike conventional CLIP-style methods that treat all non-matched pairs as strict negatives, MPDRL models sample heterogeneity using medical prior features, including disease composition, uncertainty, severity, rarity, co-morbidity, report complexity, image–report alignment difficulty, and sample representativeness. Based on these priors, MPDRL introduces difficulty-aware feature modulation, adaptive temperature adjustment, prior-soft contrastive learning, and difficulty consistency regularization to improve cross-modal representation learning.

Extensive experiments show that MPDRL performs well across multiple downstream tasks. First, in supervised linear probing, it consistently outperforms existing medical vision–language pre-training methods under different label ratios, demonstrating strong transferability and label efficiency. Second, in zero-shot classification and image–text retrieval, MPDRL achieves competitive results, showing improved cross-modal alignment and generalization. Third, in radiology report generation, it also obtains competitive performance on IU X-Ray and MIMIC-CXR. Meanwhile, ablation studies also further confirm that each component contributes consistently and complements the others.

However, MPDRL still has several limitations. First, its medical prior construction relies on disease concept extraction, uncertainty estimation, and report semantic analysis, whose quality may affect difficulty modeling. In addition, manually defined severity weights may introduce subjectivity. Second, some priors, such as image–report alignment difficulty and sample representativeness, require extra pre-computation, increasing training complexity for large-scale datasets. Finally, MPDRL has been mainly validated on chest X-rays, and its generalization to other medical imaging modalities remains to be explored.

Future work will focus on learning more robust and automatic medical-prior representations, reducing dependence on predefined disease concepts, and extending MPDRL to broader multimodal clinical scenarios.

## Data Availability

The data analyzed in this study is subject to the following licenses/restrictions: The datasets analyzed in this study are publicly available but subject to the terms and conditions of the respective repositories. Access may require registration, data use agreements, or approval from the data providers. Requests to access these datasets should be directed to MIMIC-CXR: https://physionet.org/content/mimic-cxr.
